# Completed Genome Sequences of Strains from 36 Serotypes of *Salmonella*

**DOI:** 10.1128/genomeA.01472-17

**Published:** 2018-01-18

**Authors:** James Robertson, Catherine Yoshida, Simone Gurnik, Marisa Rankin, John H. E. Nash

**Affiliations:** a National Microbiology Laboratory at Guelph, Public Health Agency of Canada, Guelph, Ontario, Canada; b National Microbiology Laboratory at Winnipeg, Public Health Agency of Canada, Winnipeg, Manitoba, Canada

## Abstract

We report here the completed closed genome sequences of strains representing 36 serotypes of *Salmonella*. These genome sequences will provide useful references for understanding the genetic variation between serotypes, particularly as references for mapping of raw reads or to create assemblies of higher quality, as well as to aid in studies of comparative genomics of *Salmonella*.

## GENOME ANNOUNCEMENT

*Salmonella* spp. are the leading cause of bacterial gastroenteritis in North America, with over 1.7 million cases per annum (1). Public health jurisdictions are replacing traditional serotyping with whole-genome sequencing (WGS) methodologies for quicker and more accurate outbreak detection and surveillance activities (2). To this end, we previously developed an *in silico* serotyping platform for *Salmonella* (3, 4).

Unfortunately, the large amount of raw data available in the SRA are primarily composed of Illumina short reads which cannot circularize the *Salmonella* genome as one contiguous nucleic acid molecule. As of November 2017, the number of fully closed genomes is 501 for *Salmonella enterica* and 4 for *Salmonella bongori*. Therefore, we sequenced 36 diverse serotypes of *Salmonella* using a combination of Illumina and PacBio technologies to produce high-quality genomes for public health and comparative genomics applications. This data set represents 25 novel serotypes with closed reference genomes.

Genomic DNA was isolated using the automated Qiagen EZ1 DNA tissue kit, using the manufacturer’s protocol, except 180 μl of G2 buffer was used with 10 μl of proteinase K and 10 μl of lysozyme (10 mg/ml; Sigma-Aldrich, Gillingham, UK). PacBio sequencing was performed at the Génome Québec Innovation Centre (McGill University, Quebec, Canada) using single-molecule real-time (SMRT) cells in an RSII sequencer, which produced 100,000 to 150,000 reads per sample, with an average read length of 6,000 bp. The PacBio read sets were assembled into circular consensus sequences using the HGAP workflow 1.1.13. Illumina sequencing on MiSeq version 3 (600-cycle kit) using Nextera XT libraries was performed at the National Microbiology Laboratory at Winnipeg (Winnipeg, Manitoba, Canada) to a target of 60-fold coverage. The quality of the Illumina read sets was examined using FastQC (http://www.bioinformatics.babraham.ac.uk/projects/fastqc/). Illumina read correction was performed using Lighter version 1.1.1 (https://github.com/mourisl/Lighter). Corrected Illumina reads were then mapped to the PacBio assembly using Bowtie2 version 2.1.0 (http://bowtie-bio.sourceforge.net/bowtie2/index.shtml) using the very-sensitive-local option. The output was sorted and converted into a bam file using SAMtools version 1.3 (http://samtools.sourceforge.net/) and input to Pilon version 1.2.2 (https://github.com/broadinstitute/pilon). The process was performed iteratively on the corrected assemblies until no changes were made to the output. Final assemblies were examined using Gap5 software version 1.2.14 (http://www.sanger.ac.uk/science/tools/gap5). Completed assemblies were processed through the *Salmonella In Silico* Typing Resource (SISTR) (3, 4) to confirm that the *in silico* predictions matched the serotype previously performed by our OIE Reference Laboratory for Salmonellosis in Guelph, Ontario, Canada.

Closed reference genomes provide great value to an understanding of the biology of pathogens, and as such, it is important that genome repositories contain as many of them as possible. These would make important contributions as reference sequences for the WGS assembly of isolates of the same or highly similar serotypes, as well as provide more accurate genomes for comparative and epidemiological studies on outbreak detection and surveillance of *Salmonella*.

### Accession number(s).

The genome sequences for these 36 *Salmonella* isolates have been deposited in DDBJ/ENA/NCBI under BioProject no. PRJNA294295. The GenBank accession numbers are listed in Table 1. The raw sequence data are available in the Sequence Read Archive.

**TABLE 1:** *Salmonella* strains sequenced in this study, by serotype

Serotype	Isolate no.	GenBank accession no.	Genome size (bp)
Antsalova	S01-0511	CP019116	4,648,086
Apapa	SA20060561	CP019403	4,801,658
Bardo	SA20113257	CP019404	4,849,139
Bergen	ST350	CP019405	4,801,835
Blegdam	S-1824	CP019406	4,693,979
Borreze	SA20041063	CP019407	4,777,558
Braenderup	SA20026289	CP022490	4,734,880
Crossness	1422-74	CP019408	4,847,468
Derby	SA20035215	CP022494	4,850,334
Djakarta	S-1087	CP019409	4,668,861
Hillingdon	N1529-D3	CP019410	4,618,056
Hvittingfoss	SA20014981	CP022503	4,940,239
India	SA20085604	CP022015	5,395,280
Johannesburg	ST203	CP019411	4,651,794
Kentucky	SA20030505	CP022500	4,782,363
Koessen	S-1501	CP019412	4,566,169
Krefeld	SA20030536	CP019413	4,942,273
Macclesfield	S-1643	CP022117	4,822,139
Manchester	ST278	CP019414	4,532,753
Manhattan	SA20084699	CP022497	4,732,484
Mbandaka	SA20026234	CP022489	4,796,292
Moscow	S-1843	CP019415	4,690,402
Nitra	S-1687	CP019416	4,691,807
Onderstepoort	SA20060086	CP022034	4,774,926
Ouakam	SA20034636	CP022116	4,874,915
Quebec	S-1267	CP022019	4,626,699
Saintpaul	SA20031783	CP022491	4,775,303
subsp. II 55:k:z_39_	1315K	CP022139	4,859,044
subsp. II 57:z_29_:z_42_	ST114	CP022467	4,719,375
subsp. IIIa 53:z_4_,z_23_,z_32_:-	SA20100345	CP022504	4,586,333
subsp. IIIb 50:k:z	MZ0080	CP022142	5,076,950
subsp. IIIb 65:c:z	SA20044251	CP022135	4,913,978
subsp. V 66:z_41_:-	SA19983605	CP022120	4,468,959
Wandsworth	SA20092095	CP019417	4,916,040
Waycross	SA20041608	CP022138	4,812,886
Yovokome	S-1850	CP019418	4,640,929
