# Correction for Robertson et al., “Completed Genome Sequences of Strains from 36 Serotypes of Salmonella”

**DOI:** 10.1128/genomeA.00333-18

**Published:** 2018-04-19

**Authors:** James Robertson, Catherine Yoshida, Simone Gurnik, Marisa Rankin, John H. E. Nash

**Affiliations:** a National Microbiology Laboratory at Guelph, Public Health Agency of Canada, Guelph, Ontario, Canada; b National Microbiology Laboratory at Winnipeg, Public Health Agency of Canada, Winnipeg, Manitoba, Canada

## AUTHOR CORRECTION

Volume 6, no. 3, e01472-17, 2018, https://doi.org/10.1128/genomeA.01472-17. Page 1: The article byline should read as given above.

Page 2, line 2 of Acknowledgments: “and Marisa Rankin for her help with proofreading the assemblies” should be removed.

